# Supplementary material for: Reset First Resistive Switching in Ni1−xO Thin Films as Charge Transfer Insulator Deposited by Reactive RF Magnetron Sputtering
Source: Nanomaterials (Basel). 2022 Jun 29;12(13):2231. doi: 10.3390/nano12132231 (PMC9268175; doi:10.3390/nano12132231)
Supplement: Supplementary file 1 [file nanomaterials-12-02231-s001.zip › nanomaterials-1780050-supplementary.pdf]

nanomaterials

MDPI

Supplementary materials

Reset first resistive switching in $Ni_{1-x}O$ thin films as charge transfer insulator deposited by reactive RF magnetron sputtering

Daewoo Kim[1], Taeho Kim[1,2], Jaeyeon Kim[1] and Hyunchul Sohn[1,*]

[1] Department of Materials Science and Engineering, Yonsei University, Seoul, 03722, Korea; daewoo.kim@yonsei.ac.kr (D.W.K.); kkthh22@yonsei.ac.kr (T.H.K.); jaeyeonkim@yonsei.ac.kr (J.Y.K.)
[2] Lam research, Icheon-si, 17336, Korea
* Correspondence: hyunchul.sohn@yonsei.ac.kr (H.C.S.); Tel.: +82-2-2123-5850

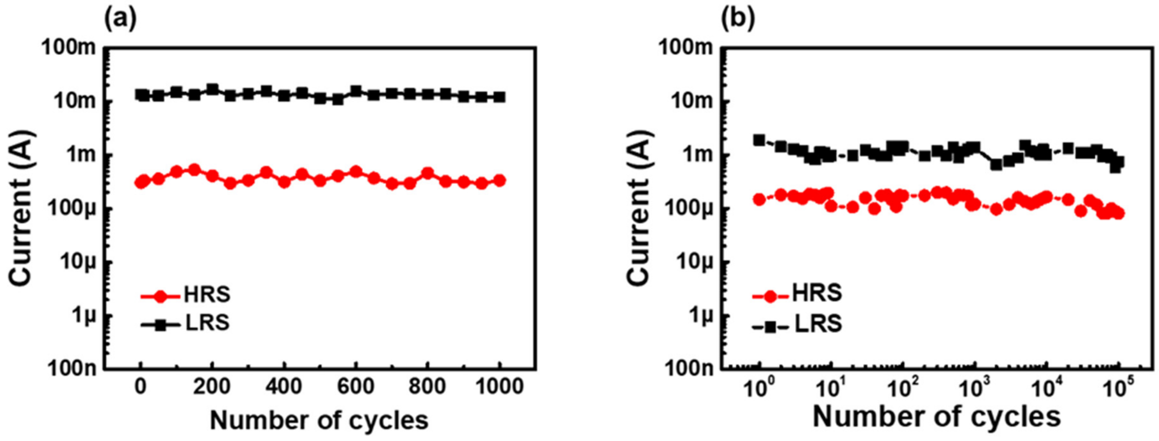

Figure S1. Endurance characteristics of $Ni_{1-x}O$ bipolar RS device with (a) DC cycles measured with V_{read} of ±0.25 V, and (b) AC pulses cycles measured with a set pulse (V_{set}) of -0.95 V with 180 ns, a reset pulse (V_{reset}) of 1.2 V with 180 ns and V_{read} of 0.3 V.
